# Effects of a 16-week multimodal exercise program on gait performance in individuals with dementia: a multicenter randomized controlled trial

**DOI:** 10.1186/s12877-020-01635-3

**Published:** 2020-07-16

**Authors:** Sandra Trautwein, Bettina Barisch-Fritz, Andrea Scharpf, Steffen Ringhof, Thorsten Stein, Janina Krell-Roesch, Alexander Woll

**Affiliations:** 1 Karlsruhe Institute of Technology, Institute of Sports and Sports Science, Engler-Bunte-Ring 15, 76131 Karlsruhe, Germany; 2 Department of Sport and Sport Science, University of Freiburg, Freiburg, Germany; 3 Department of Health Sciences Research, Mayo Clinic, Rochester, MN USA

**Keywords:** Physical activity, Neurodegenerative disorder, Walking, Physical functional performance, Cognition, Dual task

## Abstract

**Background:**

There is a high prevalence of gait impairments in individuals with dementia (IWD). Gait impairments are associated with increased risk of falls, disability, and economic burden for health care systems. Only few studies have investigated the effectiveness of physical activity on gait performance in IWD, reporting promising but inconsistent results. Thus, this study aimed to investigate the effectiveness of a multimodal exercise program (MEP) on gait performance in IWD.

**Methods:**

In this parallel-group randomized controlled trial, we enrolled 319 IWD of mild to moderate severity, living in care facilities, aged ≥ 65 years, and being able to walk at least 10 m. The control group (*n* = 118) received conventional treatment, whereas the intervention group (*n* = 201) additionally participated in a 16-week MEP specifically tailored to IWD. We examined the effects of the MEP on spatiotemporal gait parameters and dual task costs by using the gait analysis system GAITRite. Additionally, we compared characteristics between positive, non-, and negative responders, and investigated the impact of changes in underlying motor and cognitive performance in the intervention group by conducting multiple regression analyses.

**Results:**

Two-factor analyses of variance with repeated measurements did not reveal any statistically significant time*group effects on either spatiotemporal gait parameters or dual task costs. Differences in baseline gait performance, mobility, lower limb strength, and severity of cognitive impairments were observed between positive, non-, and negative responders. Positive responders were characterized by lower motor performance compared to negative and non-responders, while non-responders showed better cognitive performance than negative responders. Changes in lower limb strength and function, mobility, executive function, attention, and working memory explained up to 39.4% of the variance of changes in gait performance.

**Conclusions:**

The effectiveness of a standardized MEP on gait performance in IWD was limited, probably due to insufficient intensity and amount of specific walking tasks as well as the large heterogeneity of the sample. However, additional analyses revealed prerequisites of individual characteristics and impacts of changes in underlying motor and cognitive performance. Considering such factors may improve the effectiveness of a physical activity intervention among IWD.

**Trial registration:**

DRKS00010538 (German Clinical Trial Register, date of registration: 01 June 2016, retrospectively registered, https://www.drks.de/drks_web/setLocale_EN.do).

## Background

Gait impairments represent a major public health concern [1]. Their prevalence increases with age, and more than 32% of individuals aged 60 years and above have gait impairments [2] such as decreased walking speed, shortened stride length, and enhanced double support phase [1, 3, 4]. Gait impairments are very prevalent in individuals with dementia (IWD), with an estimated 50% of IWD being affected [5, 6]. In contrast, among cognitively unimpaired older adults, the prevalence rates range between 7 and 36% [2, 5, 6]. Dementia is an umbrella term for conditions that are characterized by a significant decline in one or more cognitive domains and behavioral changes that interfere with independence in everyday activities [7]. The causes of dementia can vary, with Alzheimer’s disease being the most common in older adults. Other dementias include vascular dementia, Lewy body dementia, or frontotemporal dementia. Furthermore, mixed dementia is common [8].

Various other motor impairments, such as reduced strength and postural control, may contribute to the increased prevalence of gait impairments in IWD [5, 9]. Moreover, gait is not merely an automated motor activity but requires input from the cerebellum, the motor cortex, and the basal ganglia, as well as an intact sensory feedback [1, 10, 11]. Thus, dementia-related pathological changes in these brain structures may also contribute to gait impairments [3].

Both gait and cognitive impairments are associated with an increased risk of falls and decreased quality of life [12, 13]. Accordingly, the incidence of falls in IWD is two to three times higher than in cognitively unimpaired older individuals [1, 5, 13]. Furthermore, the various health-related and economic consequences of falls, such as higher rates of institutionalization, disability, morbidity, mortality, and increased financial burden [1, 5], underline the need of interventions focusing on improving or maintaining gait performance in IWD. Indeed, various pharmacological and non-pharmacological interventions to improve gait performance and reduce falls in older adults have been studied [1, 5, 11, 13–16].

Physical activity interventions have shown to be effective in cognitively unimpaired older individuals and may also be beneficial for IWD [5]. However, to date, only few studies have evaluated the effectiveness of physical activity on gait performance in IWD. These studies show promising but inconsistent results. For example, seven studies observed positive effects of physical activity on walking speed as assessed through short distance walk tests [17–23], whereas fifteen studies did not report statistically significant findings [24–38]. Furthermore, ten studies applied an instrumented gait analysis, and mainly reported positive effects of physical activity on stride length [39–46], stride time [43, 45], step time [46], double support time [40], and stride frequency [41]. In contrast, no effects were found on step length [44, 47], step width [43, 47], and percent of single support [44]. Inconsistent results exist for walking speed [40–44, 46–48], stride speed [39, 45], percent of double support [43–45], and cadence [19, 39, 42, 43, 45, 46]. Findings of studies investigating dual task conditions are also inconsistent regarding potential effects on gait impairments and do not allow meaningful conclusions [26, 28, 35, 36, 39, 45]. Thus, more research is needed to better understand the potentially beneficial effects of physical activity on gait performance in IWD in both single and dual task conditions.

Most previous studies conducted multimodal physical activity interventions, including strength, balance, and aerobic exercises [17–21, 23–25, 28, 30–36, 39–45, 47]. Given the relationship between motor, cognitive and gait performance, as well as the positive impacts of cognitive training programs [1, 11], interventions combining physical and cognitive activity may be most promising for improving gait performance in IWD [15]. Indeed, studies combining physical and cognitive activity predominantly reported beneficial effects on gait performance [35, 39, 43, 45]. However, these studies had no randomized controlled trial designs [35, 39, 45], did not use instrumented gait analysis systems [35], or focused on dual task exercises while not considering other cognitive tasks [43]. This research gap emphasizes the need for additional investigations.

**Fig. 1:**
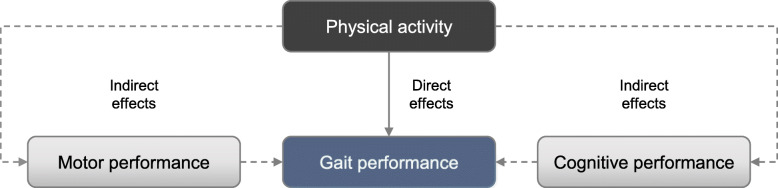
Direct and indirect effects of physical activity on gait performance

When aiming to improve the effectiveness of physical activity interventions on gait performance in IWD, it is also important to consider and identify determinants that may potentially impact the association between physical activity and subsequent changes in gait performance. However, research on such prerequisites, e.g. specific characteristics of participants that may determine which participants are most likely to benefit from specific physical activity interventions, is rare. With regard to the expected direct and indirect effects of physical activity (see Fig. 1), little is known as to how intervention-induced changes in underlying motor and cognitive performance may be related to changes in gait performance in IWD. As both other motor and cognitive impairments explain the increased prevalence of gait impairments in IWD [5], potential impacts of both factors are possible. Based on theoretical considerations, associations between changes in gait performance with changes in balance, mobility, strength and function of lower limbs [9] as well as with changes in executive function, attention, and working memory [49, 50] can be assumed.

Therefore, the primary aim of the current study was to determine the effectiveness of a multimodal exercise program (MEP), which combined both motor and cognitive tasks, on gait performance and dual task costs in IWD residing in care facilities. We hypothesized that a 16-week MEP, in addition to conventional treatment, may have a differential effect on gait performance in IWD as compared to conventional treatment alone. Our secondary aim was to identify determinants that may affect the effectiveness of the MEP, by examining differences in characteristics closely related to gait performance between positive, non-, and negative responders. Based on relations of gait performance with motor and cognitive performance, as well as the etiology of dementia [1, 3, 5, 9–11], we hypothesized that positive, non-, and negative responders of the MEP would differ in baseline performance (i.e. gait, balance, mobility, strength and function of lower limbs, executive function, attention, and working memory) and selected sample characteristics (i.e. severity of cognitive impairments, etiology of dementia, and use of walking aids). Furthermore, we also investigated impacts of intervention-induced changes in underlying motor and cognitive performance on changes in gait performance and hypothesized that changes in underlying motor (balance, mobility, strength and function of lower limbs) and cognitive performance (executive function, attention, and working memory) may have an impact on changes in gait performance in IWD who participated in a dementia-specific MEP. Due to limited prior research related to our secondary and third aim, we examined these research questions based on an exploratory analysis approach.

## Methods

For this manuscript, we followed the guidelines and recommendations of the Consolidated Standards of Reporting Trials statements [51, 52]. The reader is referred to the published study protocol for a detailed description of the study design and methods [53]. The following sections will only provide a brief summary of study methods. Further information is available in the German National Register of Clinical Trials (DRKS00010538), where we retrospectively registered this study. The ethics committee of the Karlsruhe Institute of Technology (Karlsruhe, Germany) granted ethical approval.

### Study design

We performed a multicenter, parallel group randomized controlled trial with baseline and post-intervention assessments. We allocated participants to an intervention group (IG) or control group (CG) with an allocation ratio of 2:1 using minimization software (MinimPy, Version 0.3 [54]). This allocation ratio accounts for assumed higher dropout quotes and insufficient adherence to the MEP in the IG. The randomization was carried out separately for each of the 36 care facilities that served as recruitment sites, and minimization considered age, sex, Mini-Mental State Examination (MMSE) score and performance in the modified 30-s chair stand test (30s CST) of participants (for details please refer to our study protocol [53]). If possible, we blinded investigators who examined gait, motor, and cognitive performance to group allocation, i.e., the investigators did not know whether a participant was part of the IG or CG in order to ensure objectivity of testing. However, it was not possible to blind participants. In few cases, participants themselves disclosed their group allocation to investigators.

### Participants

A power analysis (G*Power 3, Version 3.1.9.2 [55], two-factor analysis of variance (ANOVA) with repeated measurements, two groups, two measurements, α = 0.05, 1-β = 0.80, η^2^ = 0.01) revealed a required total sample size of 200 participants. Considering various potential reasons for dropout, missing data, and low adherence to the MEP, we set the sample size to 405 participants. Participants were recruited from 36 care facilities in South-Western Germany that had been contacted by the research team and asked whether they would be interested to participate in the study. Employees of the respective care facilities that were identified by the respective leadership to help with the conduct of the study identified potential eligible participants, which had to fulfill the following inclusion and exclusion criteria:

*Inclusion criteria*: a) diagnosis of dementia or “suspected” dementia (i.e. person with dementia as suspected by the treating physician based on ICD-10 criteria and MMSE performance but without a confirmed diagnosis or awaiting further clinical evaluation); b) Alzheimer’s disease, vascular dementia, or other primary dementia; c) mild to moderate severity of dementia (MMSE score: 10–24); d) age above 65 years; e) walking ability of about 10 meters with or without walking aid; and f) clearance from general practitioner.

*Exclusion criteria*: a) secondary dementia; b) other severe cognitive impairments; c) other severe neurological diseases; d) any severe acute diseases; and e) severe motor impairments.

Based on these inclusion and exclusion criteria, we verified the eligibility of participants at baseline assessment. Furthermore, we obtained written informed consent prior to the study from all participants or their legal guardians, respectively. Participation in this study was voluntary.

### Sample characteristics

The employees of the care facilities documented characteristics of participants including sex, year of birth, diagnosis of dementia, etiology of dementia, walking aids, depression, Cumulative Illness Rating Scale (CIRS) [56], and medication intake within 2 weeks of baseline assessments based on medical reports. Whenever possible, we asked physicians to retrospectively provide any missing information on their patients. In addition, we measured body weight and height in all participants.

### Intervention

Details of the intervention including examples of motor and cognitive tasks, ritualization as well as progression of exercise intensity and requirements during the course of the study are described in the study protocol [53]. Briefly, participants in the IG underwent an MEP combining motor (i.e. strength, balance, endurance, and flexibility) and cognitive tasks (i.e. memory, attention, language, and executive function). The MEP was tailored to fit the specific needs (e.g. sense of security and safety, clear verbal instruction, simplicity of tasks) and characteristics (e.g. impaired memory and attention, decreased mobility, increased fall risk) of IWD, and was delivered in the care facilities by instructors who had been specifically trained for the purpose of this study. All instructors were experienced in sports science and participated in a comprehensive training focusing on structure and contents of MEP as well as specific demands of IWD prior to delivering the intervention.

In order to provide a sense of security for participants, the MEP included a ritualization that ensured an identical sequence for all sessions. During each session, participants were asked to imagine a journey while performing appropriate motor and cognitive exercises. For example, participants performed different mobilization exercises while imagining packing their luggage as beginning ritual. The journey as well as related motor and cognitive exercises alternated throughout the course of the study. The MEP took place twice a week over a period of 16 weeks. Sessions had a duration of 60 min including about 45 min of physical exercise (the remaining 15 min were used to explain exercises, to welcome and say goodbye to participants, to answer questions or to take a short break and drink water). The MEP was delivered in a group setting (max. 12 participants, 2 instructors) and was mainly performed in a seated position with medium to submaximal intensity. Furthermore, it contained tasks in standing position and specific walking exercises. Intensity and requirements were determined on the basis of duration of exercises, number of repetitions, applied training devices, and amount of tasks in standing position as well as walking exercises. During the course of the 16 weeks, we increased the intensity of the sessions as well as the degree of motor and cognitive requirements, which were determined in the training manual. Both CG and IG participants received individually tailored conventional treatment (e.g. medication, care, or therapeutic applications) as part of standard care in their care facilities.

### Outcome measurements

We examined gait performance as outcome of interest with various spatiotemporal gait parameters of the right leg: walking speed (m/sec), stride length (cm), stride time (sec), double support phase (% of stride time), and stance phase (% of stride time). Gait analysis was performed using the electronic gait analysis system GAITRite (CIR Systems Inc., Franklin, USA, active length of 4.88 m), which has been shown to be reliable in IWD [57, 58]. All participants underwent gait analysis in single and two dual task conditions (i.e. counting backwards starting from 50 and naming animals while walking) to also assess dual tasks costs of walking while talking [59, 60]. The same dual tasks were applied for baseline and post assessment.

To eliminate acceleration and deceleration during the recording, we asked participants to start walking two meters in front of the GAITRite system and to stop walking two meters behind the system [59]. While walking at comfortable speed, participants were allowed to use walking aids as applied in everyday life. Instructions were repeated if necessary. We asked participants to repeat all conditions up to five times to generate three valid walks. Valid trials consisted of a minimum of six consecutive steps of steady-state walking, and complied with satisfactory cognitive performance in dual task conditions (i.e., stating at least three numbers in correct order and naming at least one animal, respectively). To prevent potential bias due to fatigue, participants were asked to rest between the trials if needed and we limited the assessment to five trials; thus, few participants had less than three valid trials. For statistical analysis, we considered the trial with the smallest difference to mean walking speed of all valid trials of one condition. We calculated dual-task costs using the equation $\frac{dual\ task- single\ task}{single\ task}x\ 100$ [61, 62]. Dual task costs show the percentage difference between parameters assessed during single and dual task conditions (e.g., walking speed or stride length) and thus reflect the impact of the additional cognitive task on these spatiotemporal gait parameters [61, 62].

In order to analyze differences between positive, negative, and non-responders, as well as impacts of changes in underlying motor and cognitive performance on changes in gait performance, we determined related outcomes using the motor and cognitive assessments displayed in Table 1. Experienced study staff with background in sports science supervised and administered all assessments. The reader is referred to the published study protocol [53] for a detailed description of all assessments.

**Table 1:** Motor and cognitive assessments to analyze differences between positive, non-, and negative responders, as well as impacts of changes in underlying motor and cognitive performance on changes in gait performance

Outcome	Assessment
Balance	Frailty and Injuries: Cooperative Studies of Intervention Techniques - subtest 4 (score) [63]
Mobility	Timed Up & Go Test (TUG; time in s) [64]
Strength and function of lower limb	Modified 30-Second Chair-Stand Test (30s CST; number of repetitions) [65, 66] Modified Short Physical Performance Battery (SPPB; score) [67]^a^
Global cognition	Mini-Mental State Examination (MMSE; score) [68]
Executive function and visual-spatial cognition	Clock Drawing Test (adapted Sunderland score) [69, 70]
Executive function and processing speed	Trail Making Test A (established score considering time, final number, and non-corrected mistakes, a higher score indicates better performance) [71, 72]
Attention and working memory	Digit Span forward and backward (number of correct digit spans) [73]

^a^Standing balance, gait speed, and modified 5 times sit-to-stand (with using arms)

### Statistical analysis

Statistical analysis was performed using IBM SPSS Version 25 (IBM Corporation, Armonk, USA). We ran a per protocol analysis including participants who had a MEP adherence of at least 75% (only in IG) and a complete assessment of spatiotemporal gait parameters in at least one condition of the gait assessment using GAITRite (i.e. single or two dual task conditions). Additionally, we implemented an intention-to-treat analysis and used multiple imputation (fully conditional specification imputation method, ten imputations, and ten iterations) to account for missing data. However, we did not impute data of deceased participants. To ensure plausibility of imputed data in the intention-to-treat analysis, we defined the following constraints: gait performance as both outcome and predictor variable, adherence to the MEP as well as related motor and cognitive performance as predictor variables only; minimum and maximum values according to observed range in each variable; rounding according to original data; and 100 maximal case draws, ten maximal parameter draws. We considered pooled results as provided by SPSS or reported ranges observed throughout the imputations, if SPSS did not support the pooling procedure, as final point estimates.

Required assumptions were tested before performing statistical analyses. For comparison of baseline values and sample characteristics between IG and CG, we used Chi-square tests, Mann-Whitney-U-Tests, and unpaired T-Tests according to the scaling of the investigated outcome. We analyzed treatment effects using two-factor ANOVA with repeated measurements (time*group effects), and supplemented paired T-Tests (within group time effects). A two-sided *p*-value less than 0.05 indicated statistical significance. To account for multiple comparisons, we also considered adjusted significance levels using Bonferroni-Holm correction in primary analyses. Additionally, we calculated 95% confidence intervals of differences between baseline and post-intervention assessments and partial Eta^2^.

In secondary exploratory analyses, we included walking speed, stride length, and double support of the per protocol IG sample and determined differences in baseline performance (i.e. balance, mobility, strength and function of lower limbs, executive function, attention, and working memory) and selected sample characteristics (i.e. severity of cognitive impairments, etiology of dementia, and use of walking aids) between positive, non-, and negative responders using Chi-square tests, Kruskal-Wallis-Tests, and one-factor ANOVA. For post-hoc analyses, we used Dunn-Bonferroni-Tests and Tukey-Kramer post-hoc tests, respectively. R and partial Eta^2^ served as effect sizes. We defined positive responders as those participants, who improved their gait performance at least 10% during the 16-week MEP, while negative responders showed a decline of at least 10% in gait performance, and non-responders were participants with less than 10% improvement or decline. This definition was based on percentage minimal detectable changes at 95% confidence interval of considered spatiotemporal gait parameters which ranged between 7 and 12% in a reliability study using GAITRite [58, 74]. The minimal detectable change is a measure of absolute reliability, which delineates “expected” from “true” changes in performance [57]. Moreover, we assessed the potential impact of changes in underlying motor and cognitive performance on changes in gait performance using multiple linear regression models with stepwise selection. Based on theoretical assumptions, we considered changes in balance, mobility, strength and function of lower limbs, executive function, attention, and working memory as independent variables. The calculated effect size is f^2^.

## Results

### Recruitment and flow of participants

**Fig. 2:**
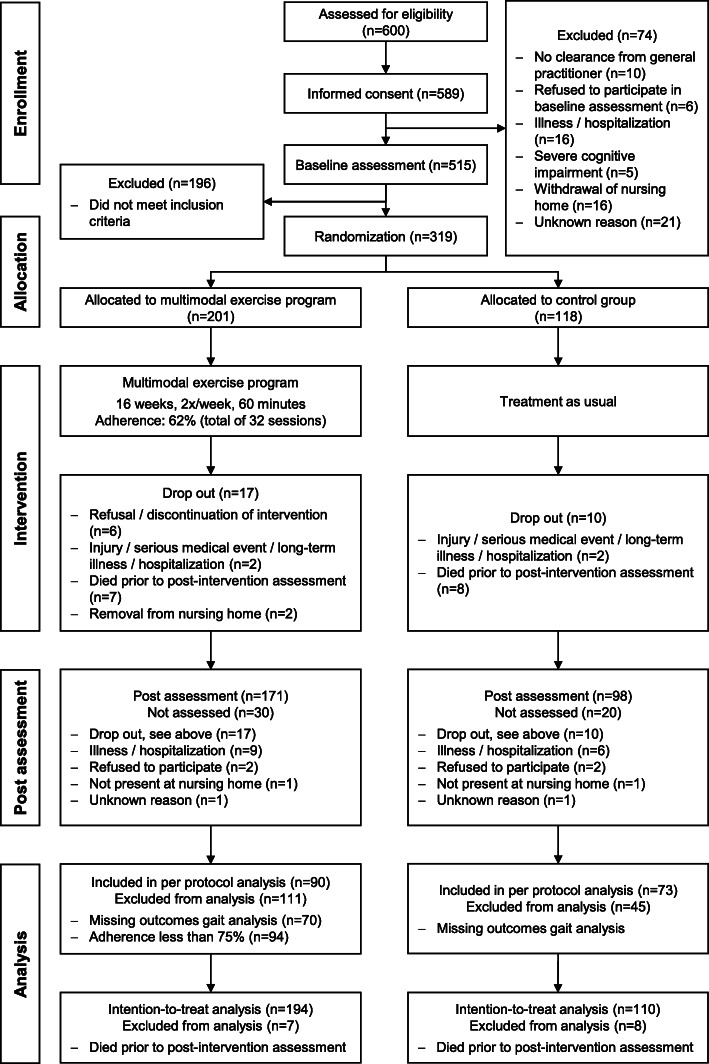
Flow of participants (n: number)

Recruitment took place between March 2015 and March 2017. We screened 600 IWD for eligibility, of whom 319 were enrolled in the study. Of these, 201 participants were allocated to the IG and 118 to the CG. There was a dropout rate of 8% in both IG and CG, respectively. Reasons for dropouts are given in Fig. 2. There were no statistically significant differences in sample characteristics or baseline measurements between participants who dropped out versus those who completed the study. The mean adherence in the IG was 62%. Overall, 107 participants (53%) of the IG completed the MEP in accordance with the study protocol, i.e. defined by a minimum adherence of at least 75% of all sessions. 65% of participants in the IG and 62% of participants in the CG completed at least one condition of gait analysis at baseline and post-intervention assessment. Based on the above-mentioned criteria, 163 participants could be considered for the per protocol analysis. Even though we extended our initially planned recruitment phase for an additional year, we were not successful in reaching our intended sample size of 405 participants. This is due to the fact that the number of participants who did not fulfill our inclusion and exclusion criteria was much larger than expected. Nevertheless, a sensitivity analysis using G*Power 3 (Version 3.1.9.2 [55]) showed that we were still able to detect small to medium effects with our actual sample size (η^2^ = 0.013 to 0.018). Figure 2 displays the flow of participants and states the reasons for dropouts and non- participations in assessments.

### Sample characteristics

Table 2 provides an overview of the characteristics of participants at baseline (per protocol analysis; see Additional file 1 for sample characteristics of the intention-to-treat analysis). High rates of dependency on walking aids and a high mean CIRS morbidity index as well as number of required medications may indicate presence of medical comorbidities in the sample. We observed no statistically significant differences in characteristics between the IG and CG, except for the number of medications.

**Table 2:** Sample characteristics of participants at baseline (per protocol analysis)

	Intervention group [*n* = 90]	Control group [*n* = 73]	Group differences [t (df)/z/Chi^2^(df), p]
**Age**, years [M (SD), range]	85 (7), 67–97	86 (5), 70–98	t(160.931) = 1.918, *p* = 0.057
**Sex**, female	82%	86%	Chi^2^(1) = 0.500, *p* = 0.479
**Type of dementia**			Chi^2^ = 5.693, *p* = 0.199
- Alzheimer’s disease	14%	15%	
- Vascular dementia	21%	11%	
- Mixed dementia	2%	4%	
- other	2%	0%	
- unknown	31%	43%	
- no confirmed/unknown diagnosis	29%	27%	
**MMSE** [M (SD), range]	17 (4), 10–24	17 (4), 10–24	t(160.446) = 0.317, *p* = 0.752
**Use of walking aid**			Chi^2^(2) = 4.644, *p* = 0.098
- walker	62%	77%	
- walking stick/s	9%	8%	
- no walking aid	29%	15%	
**CIRS** [M (SD), range]			
- Morbidity Index	9 (4), 1–20	8 (5), 2–26	t(101) = − 0.633, *p* = 0.528
- Severity Index	1.5 (0.4), 1–3 not available for 31%	1.5 (0.4), 1–3 not available for 44%	z = − 0.247, *p* = 0.805
**Number of medications** [M (SD), range]	8 (4), 1–27 unknown in 22%	5 (3), 0–12 unknown in 21%	**t(126) = −3.627,*p*** **< 0.001**
**BMI**, kg/m^2^ [M (SD), range]	28.4 (4.4), 19.7–48.5 unknown in 3%	27.1 (4.6), 17.6–36.5 unknown in 3%	t(156) = − 1.801, *p* = 0.074

*BMI*: Body Mass Index, *CIRS*: Cumulative Illness Rating Scale, *df*: degree of freedom, *M*: mean, *MMSE*: Mini-Mental State Examination, *n*: number, *SD*: standard deviation

Statistically significant results appear bold

### Effects of the multimodal exercise program on spatiotemporal gait parameters

#### Per protocol analysis

Participants of the IG (per protocol sample) had a mean adherence of 92%. Table 3 presents baseline and post-intervention values, differences between baseline and post-intervention assessments, group differences at baseline, within group time effects, and time*group effects including effect sizes of spatiotemporal gait parameters of the right leg for single and dual task conditions as well as dual task costs. We did not observe any statistically significant time*group effects. Furthermore, results on gait parameters of the left leg were comparable.

**Table 3:** Effects of the multimodal exercise program on spatiotemporal gait parameters and dual task costs (per protocol analysis)

		Baseline [M (SD)]	Group differences at baseline [t (df), p]	Post [M (SD)]	Difference post – baseline [M (SD), [CI_95_]]	Within group time effects [t (df), p]	Time*group effects	
							F (df_numerator_, df_denominator_), p	Effect size η_p_^2^
*Single task* (IG: *n* = 89, CG: n = 73)								
**Walking speed**, m/sec	IG	0.67 (0.19)	t(160) = − 1.659, *p* = 0.099	0.65 (0.22)	− 0.02 (0.13), [− 0.05, 0.00]	t(88) = 1.787, *p* = 0.077	F(1,160) = 0.036, *p* = 0.849	0.000
	CG	0.62 (0.19)		0.60 (0.20)	−0.02 (0.13), [− 0.05, 0.01]	t(72) = 1.373, *p* = 0.174		
**Stride length**, cm	IG	82.6 (19.7)	t(159.875) = −0.842, *p* = 0.401	80.5 (21.2)	−2.1 (10.9), [−4.4, 0.2]	t(88) = 1.825, *p* = 0.071	F(1,160) = 0.030, *p* = 0.863 ^a^	0.000
	CG	80.2 (15.7)		77.8 (16.9)	−2.4 (10.4), [−4.8, 0.0]	t(72) = 1.973, *p* = 0.052		
**Stride time**, sec	IG	1.3 (0.2)	**t(131.361) = 2.346,*p*** **= 0.020**	1.3 (0.2)	0.0 (0.2), [0.0, 0.1]	t(88) = − 1.571, *p* = 0.120	F(1,160) = 0.195, *p* = 0.660 ^a^	0.001
	CG	1.3 (0.2)		1.4 (0.3)	0.0 (0.2), [0.0, 0.1]	t(72) = −0.853, *p* = 0.397		
**Double support**, % of stride time	IG	38.0 (8.1)	t(160) = 1.289, p = 0.199	39.2 (8.4)	1.1 (4.9), [0.1, 2.2]	**t(88) = −2.182,*p*** **= 0.032**	F(1,160) = 0.005, *p* = 0.943	0.000
	CG	39.6 (7.4)		40.8 (7.3)	1.2 (4.9), [0.0, 2.3]	**t(72) = −2.070,*p*** **= 0.042**		
**Stance phase**, % of stride time	IG	68.9 (4.1)	t(160) = 1.368, *p* = 0.173	69.5 (4.4)	0.6 (2.5), [0.1, 1.1]	**t(88) = −2.208,*p*** **= 0.030**	F(1,160) = 0.004, *p* = 0.949	0.000
	CG	69.8 (4.1)		70.4 (4.2)	0.6 (3.1), [−0.2, 1.3]	t(72) = − 1.543, *p* = 0.127		
*Dual task, counting backwards* (IG: *n* = 62, CG: *n* = 52)								
**Walking speed**, m/sec	IG	0.55 (0.16)	**t(112) = −2.236,*p*** **= 0.027**	0.54 (0.16)	−0.02 (0.14), [− 0.06, 0.02]	t(61) = 1.001, *p* = 0.321	F(1,112) = 0.101, p = 0.752	0.001
	CG	0.48 (0.17)		0.47 (0.16)	−0.01 (0.15), [− 0.05, 0.03]	t(51) = 0.470, *p* = 0.641		
**Stride length**, cm	IG	78.2 (19.1)	t(112) = −1.407, *p* = 0.162	78.8 (19.5)	0.5 (11.6), [−2.4, 3.5]	t(61) = −0.359, *p* = 0.721	F(1,112) = 0.193, *p* = 0.661	0.002
	CG	73.5 (16.6)		75.1 (16.6)	1.6 (15.3), [−2.6, 5.9]	t(51) = −0.773, *p* = 0.443		
**Stride time**, sec	IG	1.5 (0.3)	**t(95.044) = 2.446,*p*** **= 0.016**	1.5 (0.4)	0.1 (0.4), [0.0, 0.2]	t(61) = −1.605, *p* = 0.114	F(1,112) = 0.253, *p* = 0.616 ^a, b^	0.002
	CG	1.6 (0.3)		1.7 (0.5)	0.1 (0.4), [0.0, 0.2]	**t(51) = −2.149,*p*** **= 0.036**		
**Double support**, % of stride time	IG	40.6 (9.3)	**t(112) = 2.110,*p*** **= 0.037**	41.3 (8.7)	0.7 (5.9), [−0.8, 2.2]	t(61) = −0.998, *p* = 0.322	F(1,112) = 0.042, *p* = 0.839	0.000
	CG	44.2 (9.1)		44.7 (9.0)	0.5 (7.4), [−1.6, 2.6]	t(51) = −0.481, *p* = 0.632		
**Stance phase**, % of stride time	IG	70.3 (5.2)	t(112) = 1.850, *p* = 0.067	70.4 (4.7)	0.2 (2.9), [−0.6, 0.9]	t(61) = −0.414, *p* = 0.680	F(1,112) = 0.009, *p* = 0.925 ^b^	0.000
	CG	72.1 (5.2)		72.3 (5.2)	0.2 (4.3), [−1.0, 1.4]	t(51) = −0.359, p = 0.721		
*Dual-task costs, counting backwards* (IG: n = 62, CG: n = 52)								
**Walking speed**, %	IG	−20.5 (15.2)	t(112) = −1.105, *p* = 0.271	−21.0 (15.5)	−0.6 (16.6), [−4.8, 3.6]	t(61) = 0.278, *p* = 0.782	F(1,112) = 0.053, *p* = 0.818	0.000
	CG	−23.5 (14.0)		−23.4 (17.3)	0.1 (17.3), [−4.7, 5.0]	t(51) = −0.061, *p* = 0.952		
**Stride length**, %	IG	−8.8 (11.7)	t(112) = −0.853, *p* = 0.395	−7.0 (9.4)	1.7 (13.0), [−1.6, 5.0]	t(61) = − 1.042, *p* = 0.302	F(1,112) = 0.759, *p* = 0.386 ^b^	0.007
	CG	−10.5 (9.9)		−6.6 (14.6)	3.9 (13.6), [0.1, 7.7]	**t(51) = −2.064,*p*** **= 0.044**		
**Stride time**, %	IG	17.3 (17.3)	t(112) = 0.806, *p* = 0.422	21.0 (23.9)	3.7 (25.7), [−2.8, 10.2]	t(61) = −1.130, *p* = 0.263	F(1,112) = 0.257, *p* = 0.613	0.002
	CG	19.9 (17.1)		25.9 (24.3)	6.0 (22.8), [−0.3, 12.4]	t(51) = −1.905, *p* = 0.062		
**Double support**, %	IG	11.0 (14.3)	t(112) = 0.305, *p* = 0.761	11.9 (12.8)	1.0 (15.5), [−3.0, 4.9]	t(61) = −0.491, *p* = 0.625	F(1,112) = 0.081, *p* = 0.776	0.001
	CG	11.7 (10.8)		11.8 (14.8)	0.1 (15.1), [−4.1, 4.3]	t(51) = −0.069, *p* = 0.945		
**Stance phase**, %	IG	3.1 (4.5)	t(112) = 0.095, *p* = 0.924	2.9 (3.6)	−0.2 (4.8), [−1.4, 1.1]	t(61) = 0.252, *p* = 0.802	F(1,112) = 0.130, *p* = 0.719	0.001
	CG	3.1 (3.7)		3.3 (4.8)	0.2 (5.5), [−1.3, 1.7]	t(51) = −0.254, *p* = 0.800		
*Dual task, naming animals* (IG: *n* = 61, CG: *n* = 59)								
**Walking speed**, m/sec	IG	0.45 (0.14)	t(118) = −1.797, *p* = 0.075	0.43 (0.13)	−0.01 (0.12), [− 0.04, 0.02]	t(60) = 0.805, *p* = 0.424	F(1,118) = 0.972, *p* = 0.326	0.008
	CG	0.40 (0.14)		0.41 (0.13)	0.01 (0.12), [−0.02, 0.04]	t(58) = −0.593, *p* = 0.555		
**Stride length**, cm	IG	70.4 (18.1)	t(118) = −1.415, *p* = 0.160	71.2 (17.7)	0.9 (11.0), [−2.0, 3.7]	t(60) = −0.620, *p* = 0.538	F(1,118) = 0.040, *p* = 0.841	0.000
	CG	65.9 (16.3)		66.3 (14.9)	0.4 (13.2), [−3.0, 3.9]	t(58) = −0.252, p = 0.802		
**Stride time**, sec	IG	1.6 (0.4)	t(118) = 1.480, *p* = 0.141	1.7 (0.4)	0.1 (0.3), [0.0, 0.2]	t(60) = −1.823, *p* = 0.073	F(1,118) = 3.448, *p* = 0.066	0.028
	CG	1.7 (0.5)		1.7 (0.5)	0.0 (0.3), [−0.1, 0.1]	t(58) = 0.801, *p* = 0.426		
**Double support**, % of stride time	IG	45.9 (9.4)	t(118) = 1.526, *p* = 0.130	45.2 (8.6)	−0.7 (7.1), [−2.5, 1.1]	t(60) = 0.758, *p* = 0.452	F(1,118) = 0.085, *p* = 0.771	0.001
	CG	48.5 (9.5)		48.2 (8.6)	−0.3 (7.2), [−2.2, 1.6]	t(58) = 0.326, *p* = 0.746		
**Stance phase**, % of stride time	IG	72.4 (4.7)	**t(118) = 2.233, p = 0.027**	72.3 (4.4)	−0.1 (3.9), [−1.1, 0.9]	t(60) = 0.241, *p* = 0.810	F(1,118) = 0.107, *p* = 0.744	0.001
	CG	74.5 (5.5)		74.1 (5.1)	−0.3 (3.7), [−1.3, 0.6]	t(58) = 0.727, *p* = 0.470		
*Dual-task costs, naming animals* (IG: *n* = 60, CG: n = 59)								
**Walking speed**, %	IG	−34.4 (15.9)	t(117) = −0.520, *p* = 0.604	−32.4 (18.6)	2.0 (19.7), [− 3.1, 7.1]	t(59) = − 0.776, *p* = 0.441	F(1,117) = 0.696, *p* = 0.406 ^a^	0.006
	CG	−35.9 (16.0)		−31.2 (14.6)	4.8 (16.6), [0.4, 9.1]	**t(58) = −2.204, p = 0.032**		
**Stride length**, %	IG	−17.9 (11.1)	t(117) = −0.406, *p* = 0.685	− 14.3 (12.4)	3.7 (15.4), [− 0.3, 7.6]	t(59) = − 1.842, *p* = 0.070	F(1,117) = 0.007, *p* = 0.931	0.000
	CG	−18.8 (12.5)		−14.9 (12.7)	3.9 (12.9), [0.5, 7.3]	**t(58) = −2.316,*p*** **= 0.024**		
**Stride time**, %	IG	30.1 (27.1)	t(117) = 0.326, *p* = 0.745	33.2 (29.8)	3.2 (30.3), [−4.7, 11.0]	t(59) = −0.807, *p* = 0.423	F(1,117) = 2.558, *p* = 0.112 ^b^	0.021
	CG	31.6 (24.6)		27.2 (22.7)	−4.4 (20.4), [−9.7, 0.9]	t(58) = 1.669, *p* = 0.100		
**Double support**, %	IG	25.4 (17.6)	t(117) = −0.643, *p* = 0.522	21.5 (17.8)	−3.9 (21.7), [− 9.5, 1.7]	t(59) = 1.401, *p* = 0.166	F(1,117) = 0.005, *p* = 0.946	0.000
	CG	23.4 (15.6)		19.3 (16.2)	−4.2 (15.7), [−8.3, −0.1]	**t(58) = 2.027,*p*** **= 0.047**		
**Stance phase**, %	IG	6.3 (4.5)	t(117) = 0.832, *p* = 0.407	5.5 (5.0)	−0.7 (6.0), [−2.3, 0.8]	t(59) = 0.986, *p* = 0.337	F(1,117) = 0.389, *p* = 0.534	0.003
	CG	7.0 (4.9)		5.6 (4.6)	−1.4 (4.8), [−2.6, −0.1]	**t(58) = 2.183,*p*** **= 0.033**		

Statistically significant results appear bold for α = 0.05. When considering adjusted significance levels using Bonferroni-Holm correction for multiple comparisons, no statistically significant results were observed

*CG*: control group, *CI*_*95*_: 95% confidence interval, *df*: degrees of freedom, *IG*: intervention group, *M*: mean, *n*: number, *SD*: standard deviation

^a^variance homogeneity not fulfilled

^b^covariance homogeneity not fulfilled

#### Intention-to-treat analysis

Missing data analysis showed an amount of missing data ranging between 8.5% (single task condition at baseline) and 47.6% (dual task counting backwards at post-intervention assessment). With respect to gait performance, 194 of 319 records were incomplete. Reasons for missing values included not participating at post-intervention assessment (see Fig. 2), weak physical condition, medical constrains, refusal, discontinuation of the assessment, invalid gait or dual task performance, and technical problems. Participants with incomplete data showed lower cognitive, motor, and gait performance, were older, required more medication, and had worse CIRS scores, depending on walking condition and time of assessment. Accordingly, we assumed missing at random situation, which is necessary to apply multiple imputation.

Findings of the intention-to-treat analysis were comparable to those shown in the per protocol analysis, i.e. we did not observe any statistically significant time*group effects. Please refer to Additional file 2 for results of the intention-to-treat analysis.

### Differences in characteristics between positive, negative, and non-responders (intervention group, per protocol analysis)

When taking into account walking speed, stride length, and double support in all three walking conditions, between 10 and 39% of participants in the IG improved their gait performance by at least 10% (considered as positive responders). Moreover, 23 to 61% of IG participants did not change their gait performance (considered as non-responders), while 19 to 39% showed a decline in gait performance by at least 10% (considered as negative responders). Table 4 displays the proportion of positive, non-, and, negative responders in the IG depending on spatiotemporal gait parameter and walking condition, as well as mean changes in gait performance.

**Table 4:** Positive, non-, and negative responders in the intervention group and mean changes in gait performance (per protocol analysis)

	All		Negative responders		Non-responders		Positive responders	
	n	Mean change (SD)	%	Mean change (SD)	%	Mean change (SD)	%	Mean change (SD)
Single task								
**Walking speed**, m/sec	89	−0.03 (0.21)	35%	−0.22 (0.09)	48%	−0.01 (0.05)	17%	0.32 (0.19)
**Stride length**, cm	89	−2.07 (14.98)	26%	−19.59 (10.15)	57%	−0.75 (5.42)	17%	20.30 (9.81)
**Double support**, % of stride time	89	3.57 (12.58)	29%	18.99 (8.62)	61%	−0.57 (4.69)	10%	−16.15 (6.08)
Dual task, counting backwards								
**Walking speed**, m/sec	62	0 (0.26)	39%	−0.26 (0.11)	23%	−0.02 (0.04)	39%	0.27 (0.14)
**Stride length**, cm	62	2.06 (17.08)	19%	−18.36 (7.05)	50%	−2.18 (4.88)	31%	21.87 (13.99)
**Double support**, % of stride time	62	3.16 (14.32)	29%	20.67 (9.37)	55%	−0.36 (5.56)	16%	−16.44 (4.76)
Dual task, naming animals								
**Walking speed**, m/sec	61	0.02 (0.29)	34%	−0.27 (0.12)	31%	−0.01 (0.06)	34%	0.33 (0.23)
**Stride length**, cm	61	3.49 (19.36)	23%	−18.66 (7.34)	49%	0.94 (6.27)	28%	26.21 (17.33)
**Double support**, % of stride time	61	−0.14 (14.68)	23%	21.12 (9.04)	56%	−1.99 (4.65)	21%	−18.20 (6.27)

*n*: number, *SD*: standard deviation

Positive, non-, and negative responders differed statistically significantly in terms of baseline performance of walking speed (both dual tasks), stride length (single task, dual task naming animals), double support (single task, both dual tasks), Timed Up & Go Test (TUG; single task: stride length, dual task naming animals: walking speed), modified 30s CST (single task: double support), MMSE (single task), and proportion of walking aids (dual task naming animals: stride length; see Table 5, Additional file 3 presents statistically non-significant results).

**Table 5:** Statistically significant differences in baseline motor and cognitive performance as well as the use of walking aids between positive, non-, and negative responders in the intervention group (per protocol analysis)

	Negative responders	Non-responders	Positive responders	Between group difference	Post-hoc analysis
	Mean (SD)	Mean (SD)	Mean (SD)	F (df_numerator_, df_denominator_)/ Chi^2^(df), p	
*Single task, walking speed*					
MMSE (n = 89)	14.8 (4.0)	18.5 (3.9)	16.7 (5.1)	Chi^2^(2) = 12.093, *p* = 0.002	z = −3.472, *p* = 0.002, r = 0.404 ^a^
*Single task, stride length*					
Stride length, cm (n = 89)	80.3 (19.9)	89.4 (15.2)	62.7 (20.0)	F(2,86) = 14.129, p < 0.001, η_p_^2^ = 0.247	*p* = 0.008, MD = -17.60, CI_95_ [−31.32, − 3.89] ^b^ p < 0.001, MD = -26.79, CI_95_ [− 38.93, −14.65] ^c^
TUG, sec (n = 89)	22.9 (10.9)	19.0 (7.6)	31.5 (20.0)	Chi^2^(2) = 8.234, p = 0.016	z = −2.800, *p* = 0.015, r = 0.325 ^c^
MMSE (n = 89)	14.5 (3.5)	17.8 (4.3)	17.4 (5.2)	Chi^2^(2) = 9.510, *p* = 0.009	z = −3.046, *p* = 0.007, r = 0.354 ^a^
*Single task, double support*					
Double support, % of stride time (n = 89)	36.2 (6.9)	37.4 (7.3)	47.3 (10.4)	F(2,86) = 7.721, *p* = 0.001 η_p_^2^ = 0.152	p = 0.001, MD = 11.09, CI_95_ [4.13, 18.05] ^b^ *p* = 0.001, MD = 9.89, CI_95_ [3.41, 16.37] ^c^
Modified 30s CST (*n* = 77)	7.5 (3.3)	9.0 (3.7)	4.8 (1.7)	F(2,74) = 4.508, *p* = 0.014, η_p_^2^ = 0.109	p = 0.020, MD = -4.14, CI_95_ [−7.73, −0.55] ^c^
MMSE (n = 89)	15.1 (3.9)	17.9 (4.3)	16.3 (5.4)	Chi^2^(2) = 6.742, *p* = 0.034	z = −2.558, p = 0.032, r = 0.286 ^a^
*Dual task, counting backwards, walking speed*					
Walking speed, m/sec (n = 62)	0.63 (0.17)	0.57 (0.11)	0.47 (0.15)	F(2,59) = 6.336, *p* = 0.003, η_p_^2^ = 0.177	p = 0.001, MD = -15.08, CI_95_ [−25.35, −4.81] ^b^
*Dual task, counting backwards, stride length*					
No statistically significant differences					
*Dual task, counting backwards, double support*					
Double support, % of stride time (n = 62)	37.7 (9.2)	40.0 (7.1)	47.8 (12.9)	Chi^2^(2) = 6.496, *p* = 0.039	z = −2.532, p = 0.034, r = 0.479 ^b^
*Dual task, naming animals, walking speed*					
Walking speed, m/sec (n = 61)	0.52 (0.11)	0.47 (0.13)	0.36 (0.12)	F(2,58) = 9.917, p < 0.001, η_p_^2^ = 0.255	p < 0.001, MD = -16.40, CI_95_ [−25.44, −7.35] ^b^
TUG, sec (n = 61)	18.8 (9.2)	20.8 (16.9)	24.8 (12.1)	Chi^2^(2) = 6.360, p = 0.042	n.s.
*Dual task, naming animals, stride length*					
Stride length, cm (n = 61)	77.9 (14.0)	71.8 (18.3)	61.6 (18.3)	F(2,58) = 3.596, p = 0.034, η_p_^2^ = 0.110	*p* = 0.031, MD = -16.36, CI_95_ [−31.46, −1.26] ^b^
Walking aid, % (n = 61)	85.7%	50.0%	82.4%	Chi^2^ = 7.540, p = 0.020	
*Dual task, naming animals, double support*					
Double support, % of stride time (n = 61)	41.9 (8.6)	44.7 (7.9)	53.2 (10.2)	F(2,61) = 6.570, p = 0.003, η_p_^2^ = 0.185	p = 0.003, MD = 11.35, CI_95_ [3.38, 19.32] ^b^ *p* = 0.010, MD = 8.55, CI_95_ [1.79, 15.30] ^c^

3*0s CST*: 30-s chair stand test, *CI*_*95*_: 95% confidence interval, *df*: degrees of freedom, *MD*: mean difference, *MMSE*: Mini-Mental State Examination, *n* :number, *n.s.*: not significant, *SD*: standard deviation, *TUG*: Timed Up & Go Test

^a^ post-hoc analysis: statistically significant better performance of non- compared to negative responders

^b^ post-hoc analysis: statistically significant worse performance of positive compared to negative responders

^c^ post-hoc analysis: statistically significant worse performance of positive compared to non-responders

The post-hoc analysis (see Table 5) revealed statistically significantly worse performance of positive compared to negative responders for walking speed (both dual tasks), stride length (single task, dual task naming animals), and double support (single task, both dual tasks). Besides we found worse performance of positive compared to non-responders for stride length (single task), double support (single task, dual task naming animals), TUG (single task: stride length), and modified 30s CST (single task: double support), as well as better performance of non- compared to negative responders for MMSE (single task).

### Impact of changes in underlying motor and cognitive performance on changes in gait performance (intervention group, per protocol analysis)

Several weak to moderate correlations (|*r*| = 0.248–0.436, *p* < 0.05) suggested relations of changes in underlying motor and cognitive performance with changes in gait performance in single and both dual task conditions. Multiple regression analyses revealed that changes in underlying motor and cognitive performance had an impact on changes in gait performance. Related models were statistically significant and explained 12.6 to 39.4% of the overall variance. Changes in TUG, modified 30s CST, modified Short Physical Performance Battery (SPPB), Clock Drawing Test, Digit Span forward and backward, and Trail Making Test had statistically significant regression coefficients (*p* < 0.05). Generally, improvements in gait performance were associated with improvements in motor and cognitive assessments. Table 6 presents the details of the multiple regression analysis models.

**Table 6:** Impacts of changes in underlying motor and cognitive performance on changes of gait performance (intervention group, per protocol analysis)

	B	SE	β	t	p	R^**2**^	Adjusted R^**2**^	F (df_**numerator**_, df_**denominator**_), p	f^**2**^
*Single task, changes in walking speed* (*n* = 51)									
Model						0.207	0.191	F(1,49) = 12.826, p = 0.001	0.24
Constant	−1.082	1.696		−0.638	0.526				
Changes in TUG	−0.915	0.256	−0.455	−3.581	0.001				
*Single task, changes in stride length* (n = 51)									
Model						0.146	0.128	F(1,49) = 8.352, *p* = 0.006	0.15
Constant	−2.774	1.518		−1.828	0.074				
Changes in modified SPPB	2.107	0.729	0.382	2.890	0.006				
*Single task, changes in double support* (n = 51)									
Model						0.144	0.126	F(1,49) = 8.210, p = 0.006	0.14
Constant	1.218	0.520		2.341	0.023				
Changes in modified SPPB	−0.716	0.250	−0.379	−2.865	0.006				
*Dual task, counting backwards, changes in walking speed* (*n* = 42)									
Model						0.387	0.356	F(2,39) = 12.322, p < 0.001	0.55
Constant	−5.331	1.995		−2.728	0.010				
Changes in Clock Drawing Test	3.597	0.961	0.474	3.742	0.001				
Changes in modified 30s CST	2.881	0.765	0.477	3.767	0.001				
*Dual task, counting backwards, changes in stride length* (n = 42)									
Model						0.334	0.300	F(2,39) = 9.771, p < 0.001	0.43
Constant	−0.661	1.626		−0.406	0.687				
Changes in modified SPPB	2.117	0.875	0.359	2.420	0.020				
Changes in modified 30s CST	1.519	0.716	0.314	2.122	0.040				
*Dual task, counting backwards, changes in double support* (*n* = 42)									
Model						0.438	0.394	F(3,38) = 9.871, p < 0.001	0.65
Constant	2.449	0.654		3.745	0.001				
Changes in Clock Drawing Test	−1.167	0.306	−0.469	−3.814	< 0.001				
Changes in modified 30s CST	−0.934	0.244	−0.472	−3.834	< 0.001				
Changes in Digit Span backward	−0.682	0.333	−0.250	−2.052	0.047				
*Dual task, naming animals, changes in walking speed* (*n* = 40)									
Model						0.280	0.241	F(2,37) = 7.184, p = 0.002	0.31
Constant	−1.991	1.823		−1.092	0.282				
Changes in modified 30s CST	1.796	0.706	0.364	2.544	0.015				
Changes in Trail Making Test	0.501	0.228	0.313	2.192	0.035				
*Dual task, naming animals, changes in stride length* (n = 40)									
Model						0.296	0.258	F(2,37) = 7.788, p = 0.002	0.35
Constant	−0.360	1.681		−0.214	0.832				
Changes in modified SPPB	2.790	0.781	0.500	3.571	0.001				
Changes in Clock Drawing Test	−1.774	0.785	−0.316	−2.259	0.030				
*Dual task, naming animals, changes in double support* (n = 40)									
Model						0.198	0.177	F(1,38) = 9.378, *p* = 0.004	0.22
Constant	1.426	0.927		1.538	0.132				
Changes in Digit Span forward	−1.736	0.567	−0.445	−3.062	0.004				

*30s CST*: 30-s chair stand test, *df*: degrees of freedom, *n*: number, *SE*: standard error, *SPPB*: Short Physical Performance Battery, *TUG*: Timed Up & Go Test

## Discussion

### Effects of the multimodal exercise program on spatiotemporal gait parameters

This multicenter randomized controlled trial aimed to investigate the effectiveness of a dementia-specific MEP, which combined motor and cognitive tasks, on gait performance. As we did not observe any statistically significant time*group effects, our primary hypothesis that a 16-week MEP may have a differential effect on gait performance in IWD as compared to conventional treatment alone could not be confirmed. This may be explained by the heterogeneity of the study sample or the relatively low amount of walking tasks included in the intervention.

With regard to sample characteristics as well as motor, cognitive, and gait performance at baseline, we observed large standard deviations indicating that the sample of IWD was highly heterogeneous in our study (see Tables 2 and 3, and Additional file 4). Due to this large heterogeneity, it was very difficult to adequately tailor one standardized physical activity intervention to the needs of all participants, and we postulate that there is likely no standard physical activity intervention that fits all IWD.

With respect to the applied intervention, an analysis of components of the MEP showed that it did not include a large amount of specific walking tasks. Even though we had planned to increase the number of exercises focusing on walking throughout the intervention, this was often not possible due to our principle of ensuring the safety of participants at all times during the MEP. Additionally, we assumed that tasks aiming to improve balance, mobility, strength and function of lower limbs may be sufficient to positively affect gait performance [75–78]. However, based on our findings, this assumption could not be confirmed. Thus, including a sufficient amount of specific walking exercises should be ensured in future physical activity interventions that aim at improving gait performance.

### Differences in characteristics between positive, negative, and non-responders and impact of changes in underlying motor and cognitive performance on changes in gait performance

Despite not having observed positive overall effects, additional analyses showed that between 61 and 81% of participants in the IG improved or maintained their gait performance after participating in the MEP. In studies among IWD, who usually experience rapid decline of motor, cognitive, and gait performance [8], even maintaining the current levels of performance is indicative of a beneficial effect. In order to better understand the prerequisites and impacts to induce such benefits from physical activity interventions, we conducted secondary analyses that focused on examining differences of baseline performance and sample characteristics between positive, non-, and negative responders, and also considered impacts of underlying changes in motor and cognitive performance on changes in gait performance.

As compared to negative and non-responders, positive responders primarily showed lower gait performance at baseline and additionally demonstrated lower performance in single motor assessments. Moreover, non-responders were less cognitively impaired than negative responders. Accordingly, low motor and gait performance as well as increased cognitive performance appear to be prerequisites for IWD in order to benefit from the MEP. Additionally, stepwise regression analyses supported the hypothesis that changes in underlying motor and cognitive performance have an impact on changes in gait performance. Indeed, the respective statistical models explained between 12.6 and 39.4% of the overall variance.

Focusing on prerequisites related to the effectiveness of the MEP, the observed lower motor performance of positive responders compared to non- and negative responders at baseline may indicate a greater potential for performance improvements for participants who enter the intervention with lower baseline levels of motor performance. As described above, it was not always possible to include more complex walking tasks throughout our intervention. Accordingly, the requirements necessary to induce improvements may not have reached critical thresholds in all participants. Moreover, our findings support the assumption that IWD must have sufficient cognitive capacities in order for them to successfully participate in physical activity interventions. In contrast, severe cognitive impairments may affect IWD in following instructions or adequately performing exercise tasks. Individuals with more severe cognitive impairments may depend even more on specific didactic concepts.

Surprisingly, we observed a statistically significant higher cognitive performance only among non-responders and in single task conditions. Positive responders also showed higher cognitive performance than negative responders, albeit not reaching statistical significance possibly due to a relatively lower number of positive responders. When we compared cognitive performance of participants in single and dual task conditions, we observed that participants with more severe cognitive impairments were less likely to successfully perform the walking with additional dual tasks (single task: MMSE = 16.9 (4.5), 45% with MMSE < 17; dual task: MMSE = 18.4 (4.0)/17.8 (4.3) 29%/26% with MMSE < 17). Accordingly, cognitive performance of participants who completed dual task conditions was more consistent and this may explain why cognitive performance did not differ between positive, non-, and negative responders.

Stepwise regression analyses showed differential impacts of changes in underlying motor and cognitive performance, depending on spatiotemporal gait parameter and walking condition. As expected, improvements in gait performance were associated with improvements in underlying motor and cognitive performance. The observed opposite relation between stride length in dual task naming animals condition and the Clock Drawing Test requires further examination. The amount of explained variance was higher for dual task than single task conditions. In dual task conditions, changes in motor and cognitive performance were statistically significant predictors, while gait parameters in the single task condition were only affected by motor predictors. Accordingly, changes in cognitive performance may be particularly required for changes in dual task conditions, which are primarily determined by motor and cognitive demands. Dual task performance while walking is highly relevant with regard to fall prevention, and worse performance is associated with increased risk of falls [13]. Thus, fall prevention interventions should consider dual tasks and include both, motor and cognitive exercises.

At the motor level, changes in strength and function of lower limbs as well as mobility were statistically significant predictors. The related performance was assessed with modified 30s CST, modified SPPB, which considers balance, mobility, and strength, and TUG. These findings indicate that there are several motor impacts related to changes in gait performance, and further emphasize the importance of multimodal interventions. Unexpectedly, changes in balance performance were not a statistically significant predictor. However, we assessed balance only in static positions, which may have different demands as compared to dynamic balance conditions while walking [79, 80]. Moreover, the frequent use of walking aids may have eliminated the potential impact of changes in balance performance [81]. Assumptions at the cognitive level could not be made, as cognitive predictors differed across established regression models.

### Comparison with previous studies

The findings of this randomized controlled trial are not fully in line with those observed in previous studies. In contrast to previous studies, which predominantly reported positive effects for stride length and stride time in single and dual task conditions [39–46], our investigation did not confirm the effectiveness of an MEP for these spatiotemporal gait parameters. In accordance with 20 previous studies, we did not observe statistically significant effects on walking speed [24–38, 42, 44, 47, 48] and percent of double support [44, 45], while twelve others did for single [17–23, 40, 41, 43, 46] and dual task conditions [28].

These inconsistent findings may be related to different study designs, gait assessments, interventions, and sample characteristics between previous research and our study. For example, studies considering walking speed in single task condition and reporting positive effects are characterized by more walking tasks included in the intervention [17, 19–21, 23, 40, 41, 43], higher exercise intensities [19, 20, 43], participants with less cognitive impairments [19, 21, 43], worse baseline walking performance [17, 18, 22, 23, 46], better baseline walking performance [19, 20, 43], as well as assessing gait performance during fast walking [27, 29, 31, 48] compared to our study. While the first four observations reflect the results and indications of this study, better baseline walking performance may enable instructors to implement higher exercise intensities and larger amounts of specific walking tasks. Moreover, fast walking speed may be a more specific indicator for changes. However, these indications need to be considered with caution, as several impacts and the heterogeneity of previous studies hamper comparisons.

Heterogeneity is a challenge also occurring in other studies. Some differences between our study and studies showing positive effects (considering walking speed in single task condition) may be due to less cognitive impairments of participants [19, 21, 43], only including participants able to walk without walking aids [19, 40, 43, 46], hints of enhanced individual supervision [20, 21, 23, 40, 41, 46], or classification in homogeneous subgroups [17]. One may argue that the negative impact of heterogeneity is less critical in groups of participants in mild stages of dementia, as these participants are rather able to implement different instructions. Moreover, it can be assumed that participants not dependent on walking aids are less likely to fall. Accordingly, it is possible to apply the same exercise for those participants, even if they differ in their walking performance. In contrast, participants with enhanced fall risks rely on several modifications due to safety reasons. Besides these differences related to sample characteristics, an enhanced individual supervision allows the individualization of exercise tasks and adapted support. However, such concepts cannot always be transferred into practice because of limited personal resources. Accordingly, new approaches feasible in group settings are necessary, such as the classification of homogeneous subgroups.

In addition, the etiology or underlying causes of dementia, as well as frequency and duration of physical activity interventions differ considerably among previous studies and make a comparison somewhat difficult. For example, previous studies have included participants with AD, whereas other have included individuals with mixed etiologies or have not reported etiologies. Due to differences in underlying pathologic changes, various etiologies may have a differential impact on the effects of physical activity on motor or cognitive performance. In addition, duration and frequency of physical activity interventions in previous studies vary between 3 weeks and 36 months, with sessions carried out once per week to twice daily. However, most studies applied a protocol consisting of two to three sessions per week for at least 12 weeks. This is in line with recommendations of recent systematic reviews and seems to be most effective in eliciting any effects on motor and cognitive performance in IWD [82–86]. Accordingly, our intervention had a duration of 16 weeks with a frequency of two sessions per week.

To the best of our knowledge, there are no published studies that compared the characteristics of positive, non-, and negative responders or investigated impacts of changes in underlying motor and cognitive performance on changes in gait performance.

### Strengths and limitations

With this multicenter randomized controlled trial, we aimed at conducting high-quality research to investigate the effectiveness of a physical activity intervention on gait performance in IWD. The strengths of the study include the emphasis on high-quality methods and a detailed reporting of our methods and findings [53]. Of note, we had a large sample size of over 300 individuals with mild to moderate dementia, our assessments were deemed adequate for IWD by an expert panel [87], and our MEP was specifically tailored to fit the needs and characteristics of IWD (please refer to [53]).

Nevertheless, several limitations pertain. First, multimodal interventions do not allow to unambiguously draw conclusions about causality, i.e. observed effects may be related to the MEP itself, but could also be due to the group setting and thus enhanced social interaction, or additional attention that participants received from the exercise instructors. Therfore, additional control conditions such as non-exercise groups that engage in mentally stimulating or social activities such as singing or playing cards together could have helped to limit this potential bias. Second, insufficient intensity and specificity may be potential limitations of the MEP related to the primary aim to improve spatiotemporal gait parameters. Several factors, such as characteristics of participants, group setting, or field conditions did not allow us to reach higher intensity levels, a larger amount of specific walking tasks, and progression of the MEP as initially planned. Importantly, the MEP was mainly performed in a seated position which may have limited impact on gait performance albeit we chose exercises with medium to submaximal intensity. One potential strategy to overcome this limitation is to conduct exercise interventions with smaller groups to ensure safety of participants while reaching higher intensities at the same time. In line with this, the secondary analysis showed that participants who benefitted had a lower motor baseline performance. Future interventions need to better consider individual prerequisites of participants and accordingly adapt intensity, specificity, as well as progression of exercises. Third, the assessments to determine motor performance used in this study are widely used in research but have not been specifically developed for IWD. Even though we intensively discussed the adequateness of these assessments during an expert panel [87] and carefully selected the most appropriate ones, we cannot rule out the possibility that the use of existing assessments not specifically designed for IWD may have led to biased results. For example, these assessments often do not sufficiently take into account fluctuating daily forms and motivational aspects that may play a role when examining IWD. Accordingly, results could reflect unfavorable conditions, reduced motivation, or lack of interest instead of actual motor performance. Therefore, it is critically important for future research to explore tailored motor assessments for use in IWD. Finally, our study sample included participants that did not have a confirmed diagnosis of dementia, in most cases due to limited financial resources or access to diagnostic tools and assessments. This may have an impact on the interpretability of findings.

## Conclusions

This multicenter randomized controlled trial contributes to the growing body of literature that aims at improving physical activity interventions for IWD. It shows that one standardized MEP is not effective in reducing the decline in gait performance among IWD in general. However, several participants of the IG were able to improve or maintain their gait performance after undergoing the MEP. Moreover, findings of secondary analyses allow for drawing conclusions on prerequisites and required changes that may be necessary for IWD to benefit from the MEP. These factors have important implications and should thus be considered when establishing future physical activity interventions.

Our main conclusion is that it is essential to develop and provide individualized physical activity interventions for IWD, and to consider individual characteristics and needs to improve effectiveness rather than having one standardized physical activity intervention; albeit acknowledging that this is currently more applicable to a research setting and probably more challenging for clinical settings such as nursing homes. Based on observed results in responder-non-responder-analyses, we suggest tailoring physical activity interventions to baseline performance of intended outcomes and severity of cognitive impairment. To this end, we here provide preliminary criteria on how to tailor physical activity interventions to fit the specific needs of IWD. However, further investigation and refinement of these criteria is needed to better characterize different clusters of IWD. Aside from individual characteristics and needs, it is also important to consider intended purposes when establishing physical activity interventions.

Our findings indicate that physical activity interventions aiming to improve gait performance in IWD should include multimodal motor exercises (e.g. walking, strength, balance, and mobility). As changes in motor and cognitive performance are statistically significant independent predictors for changes in gait performance, both motor and cognitive tasks should be included in interventions to potentially increase the beneficial effects on gait performance and fall prevention. Linking both conclusions, individualized approaches, which include relevant contributors for improving intended outcomes, while also tailoring requirements to prerequisites and focusing on those exercises in order to improve outcomes of especially low capacity, seem to be most promising for improving the effectiveness of physical activity interventions in IWD.

## Supplementary information

**Additional file 1.**

**Additional file 2.**

**Additional file 3.**

**Additional file 4.**

## Data Availability

Due to data protection reasons of the Karlsruhe Institute of Technology (Karlsruhe, Germany), restrictions may apply to the availability of these data. If permissible, the datasets used and/or analyzed during the current study are available from the corresponding author on reasonable request.

## Abbreviations

30s CST: 30-s chair stand test
ANOVA: Analysis of variance
CG: Control group
CIRS: Cumulative Illness Rating Scale
IG: Intervention group
IWD: Individuals with dementia
MEP: Multimodal exercise program
MMSE: Mini-Mental State Examination
SPPB: Short Physical Performance Battery
TUG: Timed Up & Go Test
