# Supplementary material for: Effects of a 16-week multimodal exercise program on gait performance in individuals with dementia: a multicenter randomized controlled trial
Source: BMC Geriatr. 2020 Jul 16;20:245. doi: 10.1186/s12877-020-01635-3 (PMC7364487; doi:10.1186/s12877-020-01635-3)
Supplement: Supplementary file 1 — Additional file 1. [file 12877_2020_1635_MOESM1_ESM.pdf]

Additional file 1. Sample characteristics of participants at baseline (intention-to-treat analysis)

	**Intervention group** [n=194]	**Control group** [n=110]	**Group differences** [t(df)/z/Chi²(df), p]
**Age**, years [M (SD), range]	86 (6), 67-102	87 (6), 66-98	t(302)=1.135, p=0.257
**Sex**, female	85%	89%	Chi²(1)=1.223, p=0.269
**Type of dementia**			Chi²=9.005, p=0.050
- Alzheimer's disease	19%	14%	
- Vascular dementia	18%	10%	
- Mixed dementia	2%	4%	
- other	2%	0%	
- unknown	26%	37%	
- no confirmed/unknown diagnosis	34%	36%	
**MMSE** [M (SD), range]	17 (4), 10-24	17 (4), 10-24	t(250.853)=0.389, p=0.698
**Use of walking aid**			Chi²(2)=4.104, p=0.128
- walker	69%	75%	
- walking stick/s	4%	8%	
- no walking aid	24%	16%	
	unknown in 3%	unknown in 1%	
**CIRS** [M (SD), range]			
- Morbidity Index	9 (4), 1-20	10 (6), 2-26	t(176)=0.469, p=0.640
- Severity Index	1.6 (0.4), 1-3	1.6 (0.5), 1-3	z=-0.273, p=0.785
	not available for 37%	not available for 50%	
**Number of medications** [M (SD), range]	7 (4), 0-27<br>unknown in 22%	6 (4), 0-20<br>unknown in 25%	**t(232)=-2.686, p=0.008**
**BMI**, kg/m² [M (SD), range]	28.5 (4.7), 18.1-48.5<br>unknown in 12%	27.2 (4.8), 17.6-38.0<br>unknown in 9%	**t(268)=-2.307, p=0.022**

BMI: Body Mass Index, CIRS: Cumulative Illness Rating Scale, df: degree of freedom, M: mean, MMSE: Mini-Mental State Examination, n: number, SD: standard deviation

Statistically significant results appear bold
