# Supplementary material for: Effects of a 16-week multimodal exercise program on gait performance in individuals with dementia: a multicenter randomized controlled trial
Source: BMC Geriatr. 2020 Jul 16;20:245. doi: 10.1186/s12877-020-01635-3 (PMC7364487; doi:10.1186/s12877-020-01635-3)
Supplement: Supplementary file 2 — Additional file 2. [file 12877_2020_1635_MOESM2_ESM.pdf]

Additional file 2. Effects of the multimodal exercise program on spatiotemporal gait parameters and dual task costs (intention-to-treat analysis)

		Baseline [M (SE)]	Group differences at baseline [t(df), p]	Post [M (SE)]	Difference post – baseline [M (SE), [CI_{95}]]	Within group time effects [t(df), p]	Time*group effects	
							$F(df_{numerator}, df_{denominator})$, p	Effect size η_p^2
Single task (IG: n=194, CG: n=110)								
**Walking speed**, m/sec	IG	0.62 (0.01)	t(136275)=-0.672, p=0.502	0.61 (0.01)	-0.01 (0.01), [-0.03, 0.02]	t(121)=0.573, p=0.568	F(1,302)=0.001 to 0.395, p=0.530 to 0.977	0.000 to 0.001
	CG	0.60 (0.02)		0.60 (0.02)	0.00 (0.02), [-0.03, 0.03]	t(238)=0.095, p=0.925		
**Stride length**, cm	IG	77.7 (1.4)	t(2596)=0.553, p=0.580	77.4 (1.6)	-0.3 (1.4), [-3.0, 2.5]	t(27)=0.190, p=0.851	F(1,302)=0.001 to 2.814, p=0.094 to 0.977 [a, b]	0.000 to 0.009
	CG	78.9 (1.7)		77.6 (1.9)	-1.3 (1.7), [-4.7, 2.0]	t(72)=0.789, p=0.433		
**Stride time**, sec	IG	1.3 (0.0)	**t(1200)=1.975, p=0.049**	1.3 (0.0)	0.0 (0.0), [0.0, 0.1]	t(46)=-1.224, p=0.227*	F(1,302)=0.325 to 4.944, p=0.027 to 0.569*	0.001 to 0.016
	CG	1.4 (0.0)		1.4 (0.0)	0.0 (0.0), [-0.1, 0.0]	t(230)=0.698, p=0.486		
**Double support**, % of stride time	IG	40.0 (0.6)	t(3372)=0.327, p=0.743	40.7 (0.6)	0.7 (0.6), [-0.4, 1.8]	t(59)=-1.318, p=0.192*	F(1,302)=0.002 to 0.265, p=0.607 to 0.961 [b]	0.000 to 0.001
	CG	40.3 (0.7)		41.2 (0.8)	0.9 (0.7), [-0.4, 2.2]	t(106)=-1.314, p=0.192*		
**Stance phase**, % of stride time	IG	69.8 (0.3)	t(1915)=0.305, p=0.760	70.2 (0.3)	0.4 (0.3), [-0.1, 1.0]	t(83)=-1.612, p=0.111*	F(1,302)=0.000 to 0.149, p=0.700 to 0.988	0.000
	CG	70.0 (0.4)		70.4 (0.5)	0.5 (0.4), [-0.3, 1.3]	t(48)=-1.143, p=0.259*		
Dual task, counting backwards (IG: n=194, CG: n=110)								
**Walking speed**, m/sec	IG	0.49 (0.01)	t(792)=-1.75, p=0.079*	0.49 (0.01)	0.00 (0.02), [-0.03, 0.03]	t(24)=0.035, p=0.972	F(1,302)=0.004 to 1.043, p=0.308 to 0.948	0.000 to 0.003
	CG	0.46 (0.02)		0.47 (0.02)	0.01 (0.02), [-0.02, 0.04]	t(185)=-0.540, p=0.590		
**Stride length**, cm	IG	71.2 (1.4)		73.1 (1.6)	1.9 (1.6), [-1.4, 5.2]	t(20)=-1.194, p=0.246*		

	CG	69.8 (1.8)	t(1093)=-0.629, p=0.529	72.7 (2.1)	2.9 (2.2), [-1.5, 7.4]	t(25)=-1.328, p=0.196*	F(1,302)=0.063 to 1.212, p=0.272 to 0.802 [b]	0.000 to 0.004
**Stride time**, sec	IG	1.5 (0.0)	t(108)=1.764, p=0.081*	1.6 (0.0)	0.0 (0.0), [0.0, 0.1]	t(30)=-0.836, p=0.410*	F(1,302)=0.000 to 4.518, p=0.034 to 0.996*[a, b]	0.000 to 0.015
	CG	1.6 (0.0)		1.7 (0.1)	0.1 (0.1), [0.0, 0.2]	t(26)=-1.118, p=0.274*		
**Double support**, % of stride time	IG	43.8 (0.7)	t(212)=1.383, p=0.168	43.6 (0.8)	-0.2 (0.9), [-2.0, 1.6]	t(19)=0.237, p=0.815	F(1,302)=0.002 to 1.099, p=0.295 to 0.969	0.000 to 0.004
	CG	45.4 (0.9)		45.1 (1.0)	-0.3 (1.0), [-2.2, 1.7]	t(32)=0.275, p=0.785		
**Stance phase**, % of stride time	IG	71.7 (0.4)	t(73)=1.482, p=0.143*	71.8 (0.4)	0.1 (0.4), [-0.8, 1.0]	t(24)=-0.210, p=0.835	F(1,302)=0.035 to 2.770, p=0.097 to 0.852	0.000 to 0.009
	CG	72.7 (0.5)		72.4 (0.5)	-0.3 (0.6), [-1.5, 0.8]	t(29)=0.566, p=0.575*		
Dual-task costs, counting backwards (IG: n=194, CG: n=110)								
**Walking speed**, %	IG	-17.1 (2.0)	t(205)=1.649, p=0.101*	-15.4 (2.9)	1.7 (3.3), [-4.9, 8.4]	t(18)=-0.534, p=0.600	F(1,302)=0.032 to 2.378, p=0.124 to 0.859 [b]	0.000 to 0.008
	CG	-22.2 (2.0)		-17.8 (3.7)	4.4 (4.1), [-3.7, 12.5]	t(85)=-1.067, p=0.289		
**Stride length**, %	IG	-6.9 (1.5)	**t(93)=-2.192, p=0.031**	-3.5 (2.5)	3.4 (2.8), [-2.5, 9.4]	t(13)=-1.212, p=0.246*	F(1,302)=1.125 to 6.013, p=0.015 to 0.290*[b]	0.004 to 0.015
	CG	-11.5 (1.4)		-3.6 (3.1)	7.9 (3.4), [1.1, 14.8]	**t(22)=-2.346, p=0.028**		
**Stride time**, %	IG	17.4 (2.1)	t(92)=0.513, p=0.609	18.6 (2.4)	1.2 (2.8), [-4.4, 6.9]	t(31)=-0.444, p=0.660	F(1,302)=0.000 to 4.709, p=0.031 to 0.998*	0.000 to 0.015
	CG	18.9 (2.2)		24.3 (3.5)	5.3 (3.8), [-2.2, 12.9]	t(27)=-1.414, p=0.169*		
**Double support**, %	IG	10.4 (1.2)	t(106)=1.572, p=0.119*	8.4 (1.8)	-2.0 (2.0), [-6.1, 2.1]	t(19)=1.004, p=0.328*	F(1,302)=0.000 to 0.715, p=0.398 to 0.998 [a, b]	0.000 to 0.002
	CG	13.4 (1.6)		11.0 (2.3)	-2.4 (2.5), [-7.4, 2.6]	t(32)=0.949, p=0.350*		
**Stance phase**, %	IG	2.8 (0.4)		2.4 (0.6)	-0.5 (0.7), [-1.8, 0.9]	t(21)=0.716, p=0.482*		

	CG	4.0 (0.5)	t(39)=1.679, p=0.101*	2.9 (0.7)	-1.1 (0.9), [-3.0, 0.7]	t(28)=1.222, p=0.232*	F(1,302)=0.001 to 3.165, p=0.076 to 0.976 [b]	0.000 to 0.010
Dual task, naming animals (IG: n=194, CG: n=110)								
**Walking speed**, m/sec	IG	0.41 (0.01)	t(103)=-0.706, p=0.482	0.42 (0.01)	0.01 (0.01), [-0.02, 0.03]	t(75)=-0.554, p=0.581	F(1,302)=0.002 to 0.974, p=0.324 to 0.967	0.000 to 0.003
	CG	0.40 (0.01)		0.41 (0.01)	0.01 (0.01), [-0.02, 0.04]	t(98)=-0.846, p=0.400		
**Stride length**, cm	IG	64.9 (1.4)	t(140)=0.394, p=0.694	67.7 (1.3)	2.8 (1.4), [0.0, 5.5]	t(25)=-2.009, p=0.056*	F(1,302)=0.001 to 3.912, p=0.049 to 0.971*	0.000 to 0.013
	CG	65.8 (1.7)		66.4 (1.6)	0.6 (1.9), [-3.2, 4.5]	t(31)=-0.316, p=0.754		
**Stride time**, sec	IG	1.7 (0.0)	t(134)=1.788, p=0.076*	1.7 (0.0)	0.0 (0.0), [0.0, 0.1]	t(28)=-0.971, p=0.340*	F(1,302)=1.921 to 10.040, p=0.002 to 0.167*[a, b]	0.006 to 0.032
	CG	1.8 (0.0)		1.7 (0.1)	-0.1 (0.1), [-0.2, 0.1]	t(21)=1.003, p=0.327*		
**Double support**, % of stride time	IG	47.6 (0.7)	t(98)=0.733, p=0.465	46.8 (0.7)	-0.8 (0.7), [-2.2, 0.6]	t(51)=1.122, p=0.267*	F(1,302)=0.016 to 2.222, p=0.137 to 0.901	0.000 to 0.007
	CG	48.4 (1.0)		48.0 (0.9)	-0.4 (1.0), [-2.5, 1.7]	t(32)=0.382, p=0.705		
**Stance phase**, % of stride time	IG	73.5 (0.3)	t(79)=1.456, p=0.149*	73.2 (0.4)	-0.3 (0.4), [-1.1, 0.5]	t(37)=0.784, p=0.438*	F(1,302)=0.092 to 3.400, p=0.066 to 0.761 [a]	0.000 to 0.011
	CG	74.4 (0.5)		73.9 (0.6)	-0.5 (0.6), [-1.8, 0.8]	t(19)=0.796, p=0.436*		
Dual-task costs, naming animals (IG: n=194, CG: n=110)								
**Walking speed**, %	IG	-29.3 (2.5)	t(46)=-0.684, p=0.498	-26.7 (2.3)	2.6 (2.7), [-2.8, 8.0]	t(27)=-0.977, p=0.337*	F(1,302)=0.010 to 1.900, p=0.169 to 0.922 [a, b]	0.000 to 0.006
	CG	-31.7 (2.0)		-26.8 (4.0)	4.9 (3.9), [-2.9, 12.7]	t(37)=-1.249, p=0.220*		
**Stride length**, %	IG	-14.9 (1.7)	t(34)=-0.565, p=0.576	-10.1 (1.8)	4.8 (2.2), [0.4, 9.2]	**t(20)=-2.235, p=0.037**	F(1,302)=0.001 to 2.157,	0.000 to 0.007
	CG	-16.3 (1.6)		-11.8 (2.9)	4.5 (3.0), [-1.6, 10.5]	t(22)=-1.496, p=0.149*		

							p=0.143 to 0.980 [b]	
**Stride time**, %	IG	28.2 (2.5)	t(103)=0.120, p=0.905	28.9 (2.4)	0.7 (2.8), [-4.8, 6.3]	t(63)=-0.263, p=0.794	F(1,302)=0.014 to 3.057, p=0.081 to 0.907 [a, b]	0.000 to 0.010
	CG	28.7 (2.8)		26.3 (3.9)	-2.4 (4.2), [-10.9, 6.2]	t(16)=0.561, p=0.582*		
**Double support**, %	IG	21.0 (1.4)	t(68)=0.089, p=0.929	17.0 (1.8)	-4.0 (2.1), [-8.1, 0.1]	t(32)=1.931, p=0.063*	F(1,302)=0.013 to 2.661, p=0.104 to 0.911	0.000 to 0.009
	CG	21.2 (1.9)		18.4 (2.6)	-2.8 (3.0), [-8.9, 3.3]	t(25)=0.923, p=0.365*		
**Stance phase**, %	IG	5.4 (0.4)	t(62)=1.416, p=0.162*	4.3 (0.5)	-1.1 (0.6), [-2.3, 0.1]	t(29)=1.807, p=0.081*	F(1,302)=0.000 to 3.328, p=0.069 to 0.996 [b]	0.000 to 0.011
	CG	6.4 (0.6)		5.1 (0.8)	-1.4 (0.9), [-3.1, 0.4]	t(22)=1.601, p=0.123*		

CG: control group, CI_{95}: 95% confidence interval, df: degrees of freedom, IG: intervention group, M: mean, n: number, SE: standard error

* statistically significant in single imputations, [a] variance homogeneity not fulfilled in all imputations, [b] covariance homogeneity not fulfilled in all imputations

Statistically significant results appear bold for α=0.05. When considering adjusted significance levels using Bonferroni-Holm correction for multiple comparisons, no statistically significant results were observed.
