# Supplementary material for: Effects of a 16-week multimodal exercise program on gait performance in individuals with dementia: a multicenter randomized controlled trial
Source: BMC Geriatr. 2020 Jul 16;20:245. doi: 10.1186/s12877-020-01635-3 (PMC7364487; doi:10.1186/s12877-020-01635-3)
Supplement: Supplementary file 3 — Additional file 3. [file 12877_2020_1635_MOESM3_ESM.pdf]

Additional file 3. Differences in baseline motor and cognitive performance as well as etiology of dementia and the use of walking aids between positive, non-, and negative responders in the intervention group (statistical nonsignificant results, per protocol analysis)

	Negative responders	Non-responders	Positive responders	Between group difference
		Mean (SD)		$F(df_{numerator}, df_{denominator})$/Chi²(df), p
Single task, walking speed				
FICSIT-4 (n=88)	2.4 (1.3)	2.4 (1.4)	2.1 (1.7)	Chi²(2)=0.726, p=0.695
Walking speed, m/sec (n=89)	0.67 (0.19)	0.69 (0.18)	0.59 (0.22)	F(2,86)=1.771, p=0.176
TUG, sec (n=89)	23.3 (12.5)	20.9 (12.5)	23.4 (10.1)	Chi²(2)=1.802, p=0.406
Modified 30s CST (n=77)	7.7 (3.4)	8.7 (3.5)	7.7 (4.6)	F(2,74)=0.754, p=0.474
Modified SPPB (n=84)	6.6 (2.7)	7.2 (2.4)	6.6 (3.4)	Chi²(2)=0.694, p=0.707
Clock Drawing Test (n=81)	3.1 (1.5)	3.1 (1.2)	2.7 (1.2)	F(2,78)=0.397, p=0.674
Digit Span forward (n=87)	4.5 (1.9)	5.4 (1.6)	5.1 (1.6)	F(2,84)=2.725, p=0.071
Digit Span backward (n=86)	2.4 (1.7)	2.8 (1.8)	2.6 (1.2)	Chi²(2)=0.555, p=0.758
Trail Making Test (n=78)	23.3 (14.3)	20.1 (13.7)	21.5 (17.4)	Chi²(2)=0.783, p=0.676
Etiology, AD % (n=63)	51.9%	64.0%	90.9%	Chi²=5.159, p=0.063
Walking aid, % (n=89)	71.0%	60.5%	73.3%	Chi²(2)=1.289, p=0.525
Single task, stride length				
FICSIT-4 (n=88)	2.3 (1.3)	2.4 (1.4)	2.0 (1.8)	Chi²(2)=1.315, p=0.518
Modified 30s CST (n=77)	8.1 (3.4)	8.6 (3.9)	7.0 (2.7)	F(2,74)=0.906, p=0.409
Modified SPPB (n=84)	6.5 (2.9)	7.2 (2.5)	6.5 (2.9)	Chi²(2)=1.137, p=0.567
Clock Drawing Test (n=81)	2.8 (1.5)	3.2 (1.3)	2.5 (0.9)	F(2,78)=1.671, p=0.195
Digit Span forward (n=87)	4.6 (1.8)	5.3 (1.7)	5.1 (1.9)	F(2,84)=1.238, p=0.295
Digit Span backward (n=86)	2.3 (1.6)	2.7 (1.8)	2.9 (1.5)	Chi²(2)=0.653, p=0.721
Trail Making Test (n=78)	19.4 (13.7)	21.6 (14.8)	25.0 (14.5)	Chi²(2)=1.482, p=0.477
Etiology, AD % (n=63)	65.0%	56.3%	81.8%	Chi²=2.222, p=0.324
Walking aid, % (n=89)	60.9%	62.7%	86.7%	Chi² (2)=3.376, p=0.185
Single task, double support				
FICSIT-4 (n=88)	2.3 (1.3)	2.4 (1.4)	1.7 (1.9)	Chi²(2)=1.930, p=0.381
TUG, sec (n=89)	23.7 (10.5)	19.9 (9.7)	31.2 (22.7)	Chi²(2)=4.807, p=0.090
Modified SPPB (n=84)	6.5 (2.8)	7.3 (2.4)	5.4 (3.6)	Chi²(2)=3.029, p=0.220
Clock Drawing Test (n=81)	3.0 (1.4)	3.1 (1.3)	2.5 (1.2)	Chi²(2)=1.452, p=0.493
Digit Span forward (n=87)	4.5 (1.7)	5.3 (1.7)	5.1 (2.0)	F(2,84)=1.862, p=0.162
Digit Span backward (n=86)	2.1 (1.6)	2.9 (1.8)	2.7 (1.4)	Chi²(2)=2.861, p=0.243
Trail Making Test (n=78)	19.7 (15.2)	23.2 (13.8)	16.1 (15.4)	Chi²(2)=2.411, p=0.300
Etiology, AD % (n=63)	57.9%	63.2%	83.3%	Chi²=1.147, p=0.626
Walking aid % (n=89)	73.1%	61.1%	77.8%	Chi²=1.556, p=0.467

Dual task, counting backwards, walking speed				
FICSIT-4 (n=61)	2.8 (1.4)	2.8 (1.4)	2.2 (1.6)	$Chi^2(2)=2.368$, $p=0.306$
TUG, sec (n=62)	19.4 (8.6)	17.7 (6.1)	23.1 (15.1)	$Chi^2(2)=2.429$, $p=0.297$
Modified 30s CST (n=56)	8.9 (3.0)	8.1 (3.1)	8.1 (4.0)	$Chi^2(2)=1.667$, $p=0.434$
Modified SPPB (n=60)	7.7 (2.2)	6.9 (2.1)	7.2 (2.9)	$F(2,57)=0.544$, $p=0.584$
Counting backwards (n=62)	17.9 (8.7)	15.4 (9.0)	17.1 (7.2)	$F(2,59)=0.393$, $p=0.676$
MMSE (n=62)	19.0 (3.7)	18.3 (3.5)	18.0 (4.5)	$F(2,59)=0.384$, $p=0.683$
Clock Drawing Test (n=58)	3.4 (1.4)	3.6 (1.0)	2.8 (0.9)	$Chi^2(2)=5.333$, $p=0.070$
Digit Span forward (n=61)	5.5 (1.5)	5.0 (1.8)	5.2 (1.7)	$F(2,58)=0.431$, $p=0.652$
Digit Span backward (n=61)	3.4 (1.7)	2.4 (1.7)	2.7 (1.5)	$Chi^2(2)=3.065$, $p=0.216$
Trail Making Test (n=56)	24.0 (13.5)	20.0 (15.2)	26.1 (14.2)	$F(2,53)=0.762$, $p=0.472$
Etiology, AD % (n=41)	62.5%	36.4%	71.4%	$Chi^2=3.157$, $p=0.230$
Walking aid, % (n=62)	62.5%	64.3%	70.8%	$Chi^2=0.472$, $p=0.837$
Dual task, counting backwards, stride length				
FICSIT-4 (n=61)	2.6 (1.2)	2.5 (1.5)	2.6 (1.6)	$Chi^2(2)=0.059$, $p=0.971$
Stride length, cm (n=62)	82.7 (16.9)	81.8 (18.1)	69.5 (19.8)	$F(2,59)=3073$, $p=0.054$
TUG, sec (n=62)	18.2 (8.3)	20.1 (7.5)	22.4 (16.9)	$Chi^2(2)=0.682$, $p=0.711$
Modified 30s CST (n=56)	8.7 (3.4)	8.5 (3.1)	8.1 (3.9)	$F(2,53)=0.138$, $p=0.871$
Modified SPPB (n=60)	7.8 (2.0)	7.0 (2.3)	7.6 (3.0)	$F(2,57)=0.613$, $p=0.545$
Counting backwards (n=62)	15.3 (9.3)	17.2 (7.4)	17.8 (8.9)	$F(2,59)=0.344$, $p=0.710$
MMSE (n=62)	18.7 (4.1)	18.1 (3.8)	18.8 (4.3)	$Chi^2(2)=0.706$, $p=0.703$
Clock Drawing Test (n=58)	3.2 (0.6)	3.3 (1.4)	3.1 (1.1)	$F(2,55)=0.161$, $p=0.852$
Digit Span forward (n=61)	5.7 (2.0)	5.1 (1.6)	5.3 (1.4)	$F(2,58)=0.532$, $p=0.590$
Digit Span backward (n=61)	3.2 (1.3)	3.0 (1.9)	2.7 (1.3)	$Chi^2(2)=0.920$, $p=0.631$
Trail Making Test (n=56)	22.0 (11.7)	23.0 (14.7)	26.1 (15.0)	$F(2,53)=0.333$, $p=0.718$
Etiology, AD % (n=41)	62.5%	50.0%	69.2%	$Chi^2=1.269$, $p=0.546$
Walking aid, % (n=62)	66.7%	64.5%	68.4%	$Chi^2=0.149$, $p>0.999$
Dual task, counting backwards, double support				
FICSIT-4 (n=61)	2.6 (1.2)	2.4 (1.6)	2.9 (1.5)	$Chi^2(2)=0.915$, $p=0.633$
TUG, sec (n=62)	19.5 (8.2)	19.0 (7.1)	26.8 (22.2)	$Chi^2(2)=0.584$, $p=0.747$
Modified 30s CST (n=56)	8.3 (3.6)	8.7 (3.4)	7.3 (2.8)	$F(2,53)=0.516$, $p=0.600$
Modified SPPB (n=60)	7.2 (2.5)	7.2 (2.5)	7.7 (2.4)	$F(2,57)=0.114$, $p=0.893$
Counting backwards (n=62)	15.9 (8.2)	17.9 (8.2)	16.2 (8.2)	$F(2,59)=0.407$, $p=0.668$
MMSE (n=62)	18.8 (3.9)	17.9 (3.9)	19.6 (4.5)	$F(2,59)=0.885$, $p=0.418$
Clock Drawing Test (n=58)	3.4 (1.4)	3.2 (1.0)	3.1 (1.4)	$F(2,55)=0.304$, $p=0.739$
Digit Span forward (n=61)	5.0 (1.7)	5.4 (1.6)	5.4 (1.7)	$Chi^2(2)=2.528$, $p=0.283$
Digit Span backward (n=61)	3.1 (1.5)	2.9 (1.9)	2.9 (1.1)	$Chi^2(2)=0.260$, $p=0.878$
Trail Making Test (n=56)	19.6 (12.7)	26.3 (14.3)	22.4 (15.6)	$Chi^2(2)=2.949$, $p=0.229$
Etiology, AD % (n=41)	66.7%	52.2%	66.7%	$Chi^2=0.887$, $p=0.676$

Walking aid, % (n=62)	66.7%	64.7%	70.0%	Chi²=0.150, p>0.999
Dual task, naming animals, walking speed				
FICSIT-4 (n=61)	2.7 (1.2)	2.4 (1.6)	2.5 (1.6)	Chi²(2)=0.338, p=0.844
Modified 30s CST (n=53)	9.7 (3.3)	9.2 (4.3)	7.2 (2.9)	F(2,50)=2.518, p=0.091
Modified SPPB (n=59)	7.6 (2.5)	7.2 (2.7)	6.8 (2.4)	F(2,56)=0.542, p=0.584
Verbal fluency animals (n=61)	7.9 (3.3)	8.7 (4.3)	8.1 (3.3)	F(2,58)=0.240, p=0.787
MMSE (n=61)	17.4 (4.2)	17.7 (4.4)	18.1 (4.5)	F(2,58)=0.121, p=0.886
Clock Drawing Test (n=56)	3.3 (1.6)	3.1 (1.3)	3.0 (0.8)	F(2,53)=0.203, p=0.817
Digit Span forward (n=60)	5.2 (1.3)	5.1 (1.9)	4.9 (2.2)	Chi²(2)=0.634, p=0.728
Digit Span backward (n=60)	2.6 (1.0)	3.5 (1.9)	2.4 (1.8)	Chi²(2)=5.727, p=0.057
Trail Making Test (n=56)	22.3 (15.1)	22.5 (13.0)	20.1 (14.5)	F(2,53)=0.164, p=0.849
Etiology, AD % (n=43)	71.4%	71.4%	53.3%	Chi²=1.378, p=0.561
Walking aid, % (n=61)	61.9%	63.2%	76.2%	Chi²(2)=1.178, p=0.555
Dual task, naming animals, stride length				
FICSIT-4 (n=61)	2.1 (1.4)	2.9 (1.4)	2.3 (1.6)	Chi²(2)=3.794, p=0.150
TUG, sec (n=61)	20.8 (9.7)	20.2 (14.3)	24.3 (13.3)	Chi²(2)=3.250, p=0.197
Modified 30s CST (n=53)	9.6 (4.1)	9.1 (3.6)	7.4 (3.2)	F(2,50)=1.486, p=0.236
Modified SPPB (n=59)	6.9 (2.4)	7.7 (2.3)	6.7 (2.8)	F(2,56)=0.944, p=0.395
Verbal fluency animals (n=61)	7.9 (3.3)	8.5 (4.1)	8.1 (3.2)	F(2,58)=0.151, p=0.860
MMSE (n=61)	15.6 (4.1)	18.2 (4.1)	18.7 (4.6)	Chi²(2)=4.724, p=0.094
Clock Drawing Test (n=56)	3.1 (1.2)	3.0 (1.4)	3.3 (1.0)	F(2,53)=0.338, p=0.715
Digit Span forward (n=60)	5.1 (1.3)	5.0 (2.0)	5.2 (1.9)	Chi²(2)=0.164, p=0.921
Digit Span backward (n=60)	2.6 (1.3)	2.9 (1.9)	2.8 (1.6)	F(2,57)=0.149, p=0.862
Trail Making Test (n=56)	19.7 (13.3)	25.0 (14.0)	17.4 (13.9)	Chi²(2)=2.758, p=0.252
Etiology, AD % (n=43)	75.0%	58.3%	72.7%	Chi²=1.028, p=0.688
Dual task, naming animals, double support				
FICSIT-4 (n=61)	2.6 (1.5)	2.7 (1.4)	2.0 (1.6)	Chi²(2)=2.576, p=0.276
TUG, sec (n=61)	20.1 (10.4)	20.2 (13.2)	26.4 (14.8)	Chi²(2)=3.326, p=0.190
Modified 30s CST (n=53)	9.8 (3.6)	9.0 (3.5)	6.6 (3.6)	F(2,53)=2.483, p=0.094
Modified SPPB (n=59)	7.5 (2.7)	7.6 (2.2)	5.8 (2.9)	F(2,56)=2.366, p=0.103
Verbal fluency animals (n=61)	7.6 (2.7)	9.1 (4.0)	6.6 (2.9)	F(2,58)=2.592, p=0.084
MMSE (n=61)	16.8 (3.9)	18.3 (4.5)	17.3 (4.4)	Chi²(2)=1.889, p=0.389
Clock Drawing Test (n=56)	3.5 (1.8)	2.9 (1.2)	3.2 (0.8)	F(2,56)=0.809, p=0.451
Digit Span forward (n=60)	5.1 (1.3)	5.1 (2.1)	5.0 (1.5)	Chi²(2)=0.118, p=0.943
Digit Span backward (n=60)	2.5 (1.2)	3.2 (1.8)	2.3 (1.6)	F(2,57)=1.563, p=0.218
Trail Making Test (n=56)	21.2 (14.2)	24.3 (13.5)	15.5 (14.0)	Chi²(2)=3.505, p=0.173
Etiology, AD % (n=43)	75.0%	62.5%	63.6%	Chi²=0.453, p=0.910

Walking aid, % (n=61)	71.4%	61.8%	76.9%	Chi^2=1.042, p=0.663

30s CST: 30-second chair stand test, AD: Alzheimer's disease, df: degrees of freedom, FICSIT-4: Frailty and Injuries: Cooperative Studies of Intervention Techniques - subtest 4, M: mean, MMSE: Mini-Mental State Examination, n: number, SD: standard deviation, SPPB: Short Physical Performance Battery, TUG: Timed Up & Go Test
