# Supplementary material for: Effects of a 16-week multimodal exercise program on gait performance in individuals with dementia: a multicenter randomized controlled trial
Source: BMC Geriatr. 2020 Jul 16;20:245. doi: 10.1186/s12877-020-01635-3 (PMC7364487; doi:10.1186/s12877-020-01635-3)
Supplement: Supplementary file 4 — Additional file 4. [file 12877_2020_1635_MOESM4_ESM.pdf]

Additional file 4. Heterogeneity of sample in motor and cognitive baseline performance (per protocol)

	**Total sample**	**Intervention group**	**Control group**
**Balance**			
FICSIT-4<br>[Median (IQR), range]	2 (2), 0-5<br>(n=160)	2 (2.5), 0-5<br>(n=89)	2 (2), 0-4<br>(n=71)
**Mobility**			
TUG, sec<br>[Mean (SD), range]	22.5 (11.2), 9.0-85.0<br>(n=160)	22.3 (12.2), 9.7-85.0<br>(n=90)	22.7 (10.0), 9.0-61.0<br>(n=70)
**Lower limb strength & function**			
modified 30s CST<br>[Mean (SD), range]	8.0 (3.6), 1-19<br>(n=141)	8.1 (3.7), 1-19<br>(n=78)	7.9 (3.6), 2-17<br>(n=63)
SPPB<br>[Mean (SD), range]	6.5 (2.7), 1-12<br>(n=152)	6.8 (2.8), 1-12<br>(n=85)	6.2 (2.6), 1-12<br>(n=67)
**Executive function**			
Clock Drawing Test<br>[Mean (SD), range]	3.0 (1.5), 1-9<br>(n=152)	3.0 (1.3), 1-8<br>(n=82)	3.1 (1.7), 1-9<br>(n=70)
Trail Making Test<br>[Mean (SD), range]	21.3 (13.0), 0-48<br>(n=144)	21.5 (14.3), 0-48<br>(n=79)	21.0 (11.4), 1-43<br>(n=65)
**Attention & working memory**			
Digit Span forward<br>[Mean (SD), range]	5.0 (1.7), 1-10<br>(n=161)	5.0 (1.7), 1-10<br>(n=88)	5.0 (1.8), 1-9<br>(n=73)
Digit Span backward<br>[Mean (SD), range]	2.8 (1.7), 0-6<br>(n=160)	2.7 (1.7), 0-6<br>(n=87)	2.9 (1.7), 0-6<br>(n=73)

30s CST: 30-second chair stand test, FICSIT-4: Frailty and Injuries: Cooperative Studies of Intervention Techniques - subtest 4, IQR: interquartile range, n: number, SD: standard deviation, SPPB: Short Physical Performance Battery, TUG: Timed Up & Go Test
